# Antioxidant defense system and family environment in adolescents with family history of psychosis

**DOI:** 10.1186/1471-244X-12-200

**Published:** 2012-11-16

**Authors:** Ana Gonzalez-Pinto, Monica Martinez-Cengotitabengoa, Celso Arango, Immaculada Baeza, Soraya Otero-Cuesta, Montserrat Graell-Berna, César Soutullo, Juan Carlos Leza, Juan Antonio Micó

**Affiliations:** 1 Stanley Institute International Mood Disorders Research Center, University of the Basque Country. Centro de Investigación Biomédica en Red de Salud Mental (CIBERSAM), 03-RC-003, Hospital Santiago, Vitoria, Spain; 2 Adolescent Unit, Department of Psychiatry. Centro de Investigación Biomédica en Red de Salud Mental (CIBERSAM), Hospital General Universitario Gregorio Marañon, Madrid, Spain; 3 Department of Child and Adolescent Psychiatry and Psychology, IDIBAPS. Centro de Investigación Biomédica en Red de Salud Mental (CIBERSAM), Hospital Clinic Universitari, Barcelona, Spain; 4 Child and Adolescent Psychiatry Unit, Department of Psychiatry, Hospital Universitario Valdecilla, Santander, Cantabria, Spain; 5 Section of Child and Adolescent Psychiatry and Psychology, Hospital Infantil Universitario Niño Jesús, Madrid, Spain; 6 Child and Adolescent Psychiatry Unit, Department of Psychiatry and Medical Psychology, University of Navarra, Pamplona, Spain; 7 Departamento de Farmacología, Facultad de Medicina. Centro de Investigación Biomédica en Red de Salud Mental (CIBERSAM), Universidad Complutense de Madrid, Madrid, Spain; 8 Department of Neuroscience, Pharmacology and Psychiatry, School of Medicine. Centro de Investigación Biomédica en Red de Salud Mental (CIBERSAM), University of Cádiz, Cádiz, Spain; 9 Unidad de Investigación en Psiquiatría, Hospital Santiago Apóstol Pabellón B, 8ª planta Olaguibel 29, Vitoria, Alava, 01004, SPAIN

**Keywords:** Oxidative stress, Family environment, Psychosis, Schizophrenia, Antioxidant enzymes, Unaffected siblings

## Abstract

**Background:**

Our objective was to determine antioxidant defence activity in healthy controls (HC) and healthy unaffected second-degree relatives of patients with early onset psychosis (HC-FHP), and to assess its relationship with familiar environment measured using the Family Environment Scale (FES).

**Methods:**

We included 82 HC and 14 HC-FHP aged between 9 and 17 years. Total antioxidant status, lipid peroxidation, antioxidant enzyme activities and glutathione levels were determined in blood samples.

**Results:**

There was a significant decrease in the total antioxidant level in the HC-FHP group compared with the HC group (OR = 2.94; *p* = 0.009), but no between-group differences in the Global Assessment of Functioning (GAF) scale scores. For the FES, the HC-FHP group had significantly higher scores in the cohesion (*p* = 0.007) and intellectual-cultural dimensions (p=0.025). After adjusting for these two FES dimensions, total antioxidant status remained significantly different between groups (OR = 10.86, *p* = 0.009).

**Conclusions:**

Although causal relationships cannot be assumed, we can state that family environment is not playing a role in inducing oxidative stress in these healthy subjects. It could be hypothesized that families with affected relatives protect themselves from psychosis with positive environmental factors such as cohesion and intellectual-cultural activities.

## Background

There is abundant evidence that free radicals play an important role in membrane pathology in the central nervous system (CNS) and, although they may not be the main contributory factor, free radicals may be involved in physiopathology of many diseases including schizophrenia [1-5].

Oxidative stress is characterised by an imbalance between oxidant molecules and antioxidant defence. Several studies suggest that the brain may be particularly vulnerable to oxidative stress [6-9]. The brain accounts for only 2% of the body weight but consumes 20% of the inspired oxygen, and its membranes are especially sensitive to oxidative damage because of their high content of polyunsaturated fatty acids.

It is difficult to determine the levels of free radicals in vivo because of their short half-life, but oxidative stress can be investigated indirectly by measuring the antioxidant defence system. The primary antioxidant cellular defence is enzymatic and includes superoxide dismutase (SOD), catalase (CAT) and glutathione peroxidase (cGPx). Glutathione (GSH) is the main non-enzymatic cellular antioxidant defence and protects cells from attack by reactive oxygen species. All of these chemical species are altered in patients with schizophrenia [10-12].

Results from animal models have shown that poorer environmental conditions are associated with more oxidative stress [13], and this is probably due to the need to transfer energy towards processes with a high energy cost, such as immune function, antioxidant defence and DNA repair processes [14]. The relationship between environmental conditions and oxidative stress has not been examined in healthy humans. In a previous study, we have shown that antioxidant defence is decreased in children and adolescents with early onset first-episode psychosis [15]. If environmental factors play a role in this, antioxidant defence may also be decreased in the unaffected relatives of such patients. Our hypothesis is that healthy control subjects with a family history of psychosis will have a lower antioxidant capacity than healthy subjects without this family history, and that the antioxidant capacity will be modulated by environmental family factors.

There is considerable evidence for the hereditary nature of schizophrenia, and the risk of developing the disease is increased in close relatives of people with schizophrenia [16,17]. Oxidative stress levels are influenced by various factors, including genetics. It has been shown that antioxidant capacity could have a genetic basis [18-20], which would be modified throughout life by environmental factors [21], suggesting the possible presence of an early dysfunction in the antioxidant defence system in genetically predisposed individuals [22]. Besides, high levels of expressed emotion are associated with a predominance of positive symptoms and with a worse outcome in patients with psychosis [23].

The objectives of this study are to determine antioxidant defence at the peripheral level in healthy subjects with a family history of psychosis and to compare it with that of healthy people without affected relatives. We also examine the association between oxidative stress and familiar environment.

## Methods

### Subjects

All subjects belonged to a healthy control group described previously [24]. As the sample is part of another study measuring the interaction with family environment, we considered it important not to include the first-degree relatives of patients with psychosis otherwise the disease itself could be responsible for some of the problems present in this family environment. Briefly, the subjects were aged from 9 to 17 years and were recruited from publicly-funded schools and from children who were seen for routine paediatric visits at six university hospitals. A trained psychologist carried out a telephone screening to check for exclusion criteria and subjects who passed the initial screening were interviewed with their relatives at clinical centres by psychiatrists experienced in working with children and adolescents. The exclusion criteria were the presence of any psychiatric disorder measured using the Kiddie Schedule for Affective Disorders and Schizophrenia, Present and Lifetime version (K.SADS.PL) [25], Spanish version [26], neurological disorders, head trauma, pregnancy or mental retardation (DSM-IV criteria) [27]. Healthy control subjects who had first-degree relatives with psychotic disorders were excluded, but those with second-degree relatives with psychosis were not excluded.

The healthy control subjects included in the study were divided into two groups: healthy controls (HC) without a family history of psychotic illness (n=82) and healthy controls with second-degree relatives with psychotic disorders (HC-FHP) (n=14). The family history of psychosis was evaluated with a protocol that assessed first-, second- and third-degree family antecedents of mental illnesses, according to DSM-IV criteria. To obtain this information, we interviewed at least one adult relative of each participant if one or several members of the family had been diagnosed with a psychotic disorder with delusions or hallucinations that required psychiatric treatment, including psychotic disorder not otherwise specified, schizophreniform disorder, depressive disorder with psychotic symptoms, bipolar disorder, schizophrenia, schizoaffective disorder and other psychotic disorders. None of the healthy control subjects had families with organic psychosis or psychosis secondary to substance abuse.

The study was approved by the Ethics and Clinical Research Boards of all the Hospitals involved in the study. Parents or legal representatives gave written informed consent and participants assented to participate in the study.

### Experimental procedures

Blood samples obtained by venipuncture from study subjects were taken into heparinized tubes. Red blood cells and plasma were separated by centrifugation and immediately frozen at -80°C until analysis. Total antioxidant status (TAS) and lipid peroxidation tests were determined by standardized spectrophotometric assays in plasma. The TAS assay uses the ability of antioxidants present in plasma to inhibit oxidation of the chemical ABTS, which is monitored by reading the absorbance at 600 nm. The assay for lipid hydroperoxides (LOOHs) is based on the oxidation of ferrous ions to ferric ions by hydroperoxides under acidic conditions [28]. Enzyme activities and total GSH levels were also determined by standardized spectrophotometric assays in haemolysates of erythrocytes. Determination of total GSH was based on the formation of a chromophoric thione by specific elimination of GSH-thioether, and the absorbance measured at 420 nm is directly proportional to the GSH concentration. CAT activity was measured in two steps: first, a sample containing catalase was incubated in the presence of a known concentration of H_2_O_2_; second, the H_2_O_2_ remaining in the reaction mixture was determined by an oxidative coupling reaction catalyzed by peroxidase and the resulting quinoneimine dye was measured at 520 nm [29]. cGPx enzyme activity was determined by monitoring the oxidation of NADPH to NADPH+ by a decrease in absorbance at 340 nm [30]. The determination of SOD activity was based on the SOD-mediated increase in the rate of auto-oxidation of a tetracyclic catechol in aqueous alkaline solution to yield a chromophore with maximum absorbance at 525 nm [31]. All assays were performed in a diode-array detector coupled to a thermostatically-controlled water bath.

Sociodemographic data were collected and subject global functioning was assessed using the Global Assessment of Functioning (GAF) scale. Family environment was assessed using the FES (Family Environment Scale) [32], which consists of 90 true-false items providing scores on 10 family environment dimension subscales: cohesion, expressiveness, conflict, independence, achievement, intellectual-cultural, social-recreational, moral-religious, organization and control.

### Statistical analysis

Statistical analyses were conducted with the Statistical Package for the Social Sciences v16.0 (SPSS) and with the Statistical Package R 2.5.1. The characteristics of the two groups of subjects (HC and HC-FHP) were summarized using descriptive statistics: frequencies and percentages for categorical variables, and means and standard deviations for continuous variables. Comparisons between groups were made using the Student’s t test for continuous variables with a normal distribution and the Mann–Whitney U test when the normality hypothesis was rejected, and the chi-square test or Fisher’s exact test for categorical variables. Associations between variables were examined using Pearson′s correlations. Logistic regression models were used to assess the influence of family environment on oxidative stress.

## Results

### Sociodemographic characteristics

Sociodemographic characteristics of the two groups of healthy subjects (HC and HC-FHP) are summarized in Table 1 together with the parental socioeconomic (SES) and occupational status. Mean age of subjects was similar in the HC and HC-FHP groups (15.06 ± 2.03 and 15.86 ± 1.23 years) and there were no significant differences between groups in sex distribution, socioeconomic status, parental occupation, or tobacco use.

**Table 1:** **Sociodemographic characteristics of healthy controls** (**HC**) **and healthy controls with family history of psychosis** (**HC**-**FHP**)

	**HC**	**HC**-**FHP**
**N**	82	14
***Age****(****years****)*		
Mean	15.06	15.86
Standard deviation	2.03	1.23
Median	16	16
U Mann Whitney	460	
z	−1.214	
p	0.225	
***Gender***		
Frequency (male)	52 (63.41%)	8 (57.14%)
Chi-square	0.201	
p	0.654	
***Parental SES***		
1 (highest)	8	1
2	18	4
3	24	3
4	10	0
5	22	6
p (Fisher)	0.589	
***Parental occupational category***		
1(lowest)	2	1
2	9	2
3	24	5
4	9	0
5	10	0
6	8	0
7	3	0
8	11	4
9	6	2
p (Fisher)	0.347	
***Tobacco use***		
Frequency (smokers)	4 (49%)	2 (14,3%)
p (Fisher)	0.210	

### Antioxidant enzyme activities

Means and standard deviations of the oxidative stress variables in both groups are shown in Table 2. The total antioxidant status (TAS) level was significantly lower in the HC-FHP group compared with the HC group (OR = 2.937, *p* = 0.009).

**Table 2:** Oxidative stress variables in healthy controls (HC) and healthy controls with family history of psychosis (HC-FHP): mean and standard deviation

	**Mean (SD)**		**U**	**z**	**p**	**OR**
	**HC**	**HC-FHP**				
CAT (U/ml)	10988 (4386)	9072 (6720)	434.00	0.828	0.408	-
cGPx (mU/ml)	905 (459)	953 (1100)	381.500	1.423	0.155	-
GSH (mM)	383 (156)	433 (179)	412.00	−1.078	0.281	-
LOOH (μM)	5.97 (3.90)	7.50 (6.24)	362.00	−0.388	0.698	-
SOD (U/ml)	4038 (2936)	2618 (2653)	363.00	1.633	0.102	-
TAS (mM)	1.31 (0.47)	0.95 (0.40)	281.00	2.606	**0.009**	**2.937**

### General and family functioning

GAF scale scores did not differ between the HC and HC-FHP groups (Table 3). However, as shown in Table 3, the scores for the cohesion and intellectual-cultural dimensions of the FES were significantly higher in HC-FHP group compared with the HC group (OR = 3.824, *p* = 0.007 and OR = 6.356, *p* = 0.025, respectively), reflecting a more supportive family environment. There were no between-group differences for the other dimensions of FES.

**Table 3:** Social and family environment: mean and standard deviation of the scores in GAF and FES dimensions

	**Mean (SD)**		**U**	**z**	**p**	**OR**
	**HC**	**HC-FHP**				
**GAF**	91.98 (4.46)	91.86 (2.91)	544	0.322	0.747	
**FES Dimensions**						
Cohesion	50.57 (9.20)	56.50 (5.64)	319	−2.709	**0.007**	**3.824**
Expressiveness	50.06 (8.86)	51.93 (8.27)	499	−0.793	0.428	
Conflict	45.77 (8.16)	44.21 (5.99)	530	0.473	0.636	
Independence	46.39 (7.60)	44.93 (5.34)	486	0.936	0.349	
Achievement	48.49 (7.41)	50.14 (7.11)	488	−0.909	0.363	
Intellectual-cultural	53.91 (9.98)	59.86 (5.70)	360	−2.243	**0.025**	**6.356**
Social-recreative	55.84 (8.75)	57.57 (6.64)	516	−0.606	0.545	
Moral-religious	46.68 (9.82)	45.43 (8.42)	560	0.148	0.882	
Organization	50.76 (10.11)	54.29 (5.78)	490	−0.885	0.376	
Control	50.57 (8.50)	50.29 (8.22)	572	−0.016	0.987	

After adjusting for these statistically significant positive FES dimensions in the logistic regression model, total antioxidant status remained significantly different between the two groups of healthy subjects: OR= 10.86, 95% CI= 1.82−64.74, *p*= 0.009. There was no significant correlation between FES dimensions and total antioxidant status.

## Discussion

Our results show that the HC-FHP group had a lower total antioxidant status than the HC group without a family history of psychosis, but the significant difference in antioxidant status was not mediated by negative family environmental factors. Psychosis has been associated with negative family environmental factors and increased expressed emotions [33,34]. In our study, however, the HC-FHP group (who had second-degree relatives with a psychotic episode) did not share these negative family environmental factors. Moreover, there were some environmental factors that were more positive in HC-FHP group than in the HC group. Although our results do not imply causal relationships, we can state that family environment is not a factor in inducing oxidative stress in these healthy subjects. Indeed, it could be hypothesized that families with psychotic relatives may protect themselves with positive environmental factors, such as cohesion and intellectual-cultural activities. Previously, it was found that close relatives of patients with schizophrenia had lower levels of SOD and CAT (similar to patients) and higher levels of cGPx than healthy control subjects. In our sample, although the difference was non-significant, there were numerically higher levels of cGPx in the HC-FHP group than in the HC group. Increase in oxidative stress can reflect, as stated before [35], a vulnerability to psychosis. In their study, Ben et al. [35] considered that the higher cGPx activity found in their sample of unaffected siblings could protect them against brain damage induced by free radicals. cGPx provides an effective protective mechanism against cytosolic injury because it eliminates H_2_O_2_ and lipid peroxides [36]. In our study, we found that another factor, family environment, may be a possible mediator of protection against psychosis in vulnerable people. Taken together, these findings suggest that there is an increase of oxidative stress in families at risk of psychosis that is independent of familiar environmental factors.

### Limitations

Our study has several limitations that need to be considered. First, the small sample size may obscure some of the associations found. Second, our study population consisted of healthy volunteers, which may limit the generalizability of the findings. Finally, we used a cross-sectional design and, therefore, could not examine direct causative mechanisms or the effects of progression of illness.

## Conclusions

Although further studies are warranted, the results of our study in healthy subjects with and without a family history of psychosis suggest that family environment may be a possible mediator of protection against psychosis in vulnerable people (i.e. those with affected relatives). Overall, our findings show that there is an increase of oxidative stress (reduced antioxidant defence) in families at risk of psychosis, which is independent of familiar environmental factors.

## Abbreviations

CNS: Central Nervous System; SOD: Superoxide dismutase; CAT: Catalase; cGPx: Glutathione peroxidase; HC: Healthy controls; HC-FHP: Healthy controls with family history of psychosis; TAS: Total antioxidant status; LOOHs: Lipid hydroperoxides; GSH: Glutathione.

## Competing interests

The authors report no conflict of interest in connection with the manuscript.

## Authors’ contributions

AG-P, JAM and MM-C designed the study, wrote the protocol and prepared the manuscript. CA, IB and SO-C managed the literature searches. All authors contributed in data collection. MG-B and CS contributed to the analysis and interpretation of data. JCL reviewed the manuscript. All authors have approved the final manuscript.

## Pre-publication history

The pre-publication history for this paper can be accessed here:

http://www.biomedcentral.com/1471-244X/12/200/prepub
